# *XPF*-673C>T Polymorphism Effect on the Susceptibility to Esophageal Cancer in Chinese Population

**DOI:** 10.1371/journal.pone.0094136

**Published:** 2014-04-07

**Authors:** Yingwen Liu, Lei Cao, Jiang Chang, Jia Lin, Bing He, Juan Rao, Zhi Zhang, Xuemei Zhang

**Affiliations:** 1 Department of Molecular Genetics, College of Life Science, Hebei United University, Tangshan, China; 2 Department of Epidemiology, College of Public Health, Hebei United University, Tangshan, China; 3 Department of Etiology and Carcinogenesis, Cancer Institute and Hospital, Chinese Academy of Medical Sciences, Beijing, China; 4 Hebei United University Affiliated Tangshan Gongren Hospital, Tangshan, China; MOE Key Laboratory of Environment and Health, School of Public Health, Tongji Medical College, Huazhong University of Science and Technology, China

## Abstract

**Purpose:**

Xeroderma pigmentsum group F (XPF) plays a pivotal role in DNA nucleotide excision repair and has been linked to the development of various cancers. This study aims to assess the association of *XPF* genetic variants with the susceptibility to esophageal squamous cell carcinoma (ESCC) in Chinese population.

**Methods:**

This two-stage case-control study was conducted in a total of 1524 patients with ESCC and 1524 controls. Genotype of *XPF* -673C>T and 11985A>G variants were determined by polymerase chain reaction-based restriction fragment length polymorphism (PCR-RFLP). Logistic regression analysis was performed to estimate odd ratios (ORs) and 95% confidence intervals (95% CI).

**Results:**

Our case-control study showed that *XPF* -673TT genotype was associated with a decreased risk of ESCC compared with CC genotype in both case-control sets (Tangshan set: OR = 0.58; 95%CI = 0.34–0.99, *P* = 0.040; Beijing set: OR = 0.66; 95%CI = 0.46–0.95, *P* = 0.027). Stratified analyses revealed that a multiplicative interaction between -673C>T variant and age, sex or smoking status was evident (Gene-age: P_interaction_ = 0.002; Gene-sex: P_interaction_ = 0.002; Gene-smoking: P_interaction_ = 0.002). For *XPF* 11985A>G polymorphism, there was no significant difference of genotype distribution between ESCC cases and controls.

**Conclusion:**

These findings indicated that genetic variants in *XPF* might contribute to the susceptibility to ESCC.

## Introduction

Esophageal squamous cell carcinoma (ESCC), as one of the most common malignant tumors, is a serious threat to human and health. Almost 50% of ESCC cases occur in China [1].The development of ESCC is a complex process, which is related to the multiple environment factors, including diet [2], infection [3], lifestyle factors, particularly tobacco smoking and alcohol [4]. However, individuals, who exposed to the same risk factors, had different susceptibility to ESCC, indicating the essential role of genetic factor in the development of ESCC [5], [6], [7].

Nucleotide excision repair (NER) was one of the most versatile DNA repair systems. It removes a wide range of DNA lesions, such as UV-included pyrimidine dimer, DNA cross-link and oxidative damage to maintain DNA stability [8], [9]. Deficiencies in the DNA repair capacity have been linked to increased risk of multiple cancers [10].

Xeroderma pigmentsum group F (XPF), as one of essential NER proteins [10], formed a tight complex with excision repair cross complementation 1 (ERCC1) to excise the damaged DNA [11], [12]. An *in vitro* study demonstrated that *XPF*-673C>T variant changed the transcriptional activity of gene [13]. Epidemiological studies also showed that *XPF* genetic variants contributed to the susceptibility to various cancers, such as bladder, breast, lung and gastric cancer [14], [15], [16], [17].

Considering the pivotal role of *XPF* in NER, we supposed that *XPF* polymorphisms contributed to the risk of developing ESCC. To verify this hypothesis, we carried out this case-control in a Chinese population.

## Materials and methods

### Study subject and ethics statement

In this study, two independent case-control sample sets were used. (a) Tangshan case-control set: 500 patients with ESCC recruited from Tangshan Gongren hospital (Tangshan, China) between March 2008 and December 2012 and 500 cancer-free controls. (b) Beijing case-control set: 1024 ESCC patients recruited from Cancer Hospital of the Chinese Academy of Medical Sciences (Beijing, China) between January 2009 and December 2012 and 1024 healthy controls. All the participants were genetically unrelated Han Chinese. The eligible patients were primary histopathologically confirmed and previously untreated by radiotherapy and chemotherapy. There were no age, gender, stage, or histology restrictions. Patients with previous malignancy or metastasized cancer from other organs were excluded. The controls were randomly selected from cancer-free population from the community conducted in the same region during the same period when patients were recruited. The selection criteria for the controls included no prior history of malignancy, and control subjects were frequency-matched to the patients by age (±5 years) and gender. At recruitment, written informed consent was obtained from each subject. This study was approved by the Institutional Review Board of Hebei United University and Chinese Academy of Medical Sciences Cancer Institute.

### XPF genotyping

The genotypes of *XPF*-673C>T (rs3136038) and 11985A>G (rs254942) polymorphisms were determined by polymerase chain reaction based restriction fragment length polymorphism (PCR-RFLP). *XPF* PCR fragments containing -673 C>T or 11985A>G site were amplified with the primers pairs of XPF-673F (5′ - GGG AGG CAA ACA GAG GTC TGA ATT - 3′)/XPF-673R (5′-TGC GAT TAC TCC CCA TCC TTC TT- 3′) or *XPF* -11985F(5′-GGA GTC AAG AAA CAG CCA ACC TAG TA-3′)/*XPF*-11985R (5′-AGG AAG ACA GGA TGA CAG CCA G-3′). PCR was performed in a 25 ul reaction mixture containing 10 ng DNA, 0.3 μmol each primer, and 2.5 mM MgCl_2_, 1.25 mM dNTPs, 1.5 U DNA Taq DNA polymerase. The reaction was accomplished with a profile consisting of an initial melting step of 2 min at 95°C, followed by 35 cycles of 30 s at 94°C, 30 s at 58°C, 45 s at 72°C, and a final elongation step of 72°C for 10 min. The amplified PCR products were 137 bp and 129 bp for -673C>T and 11985A>G, respectively.

For -673C>T, PCR products were digested by EcoR *I*. The restriction product was visualized on 3.5% agarose gel. The -673C allele generated 114-bp and 23-bp two bands; the -673T allele produced a single 137-bp band. For 11985A>G variant, PCR products were digested by Rsa *I* and then separated on 3.5% agarose gel. The 11985G allele generated 104-bp and 25-bp two bands and the 11985A allele produced a single 129-bp band.

**Figure 1:**
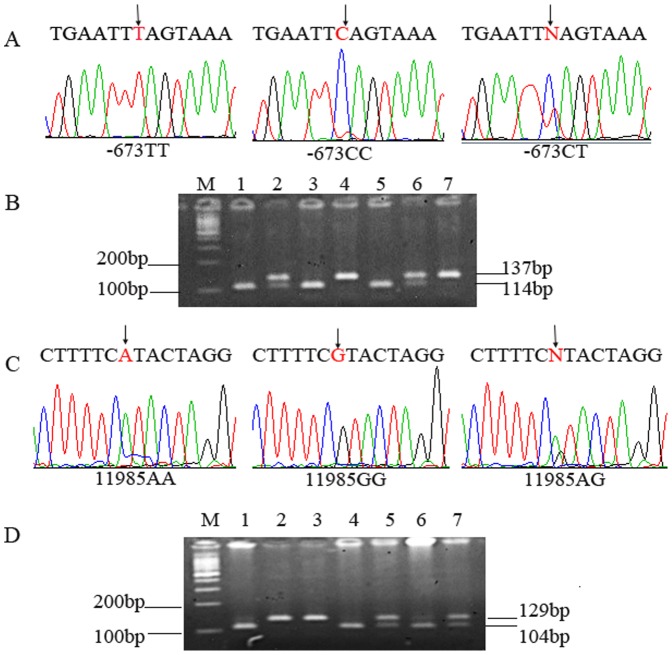
Sequencing and PCR-RFLP analysis of *XPF* polymorphisms. Figure A and C present sequencing pictures of genotypes of *XPF* -673 C>T and 11985A>G; Figure B presents the results of PCR-RFLP analysis of -673C>T polymorphism for representative cases (M: DNA marker; Cases 1, 3 and 5: CC genotype; Case 2 and 6: CT genotype; Case 4 and 7: TT genotype); Figure D presents the results of PCR-RFLP analysis of 11985A>G polymorphism for representative cases (M: DNA Marker; Case 1, 4 and 6: GG genotype; Case 5 and 7: AG genotype; Case 2 and 3: AA genotype).

The genotypes distinguished by PCR-RFLP were further confirmed by direct sequencing (Figure 1). Genotyping was performed without knowledge of the case/control status of the study subjects. A 10% masked random samples were tested by different persons and the results were all concordant.

### Statistical analysis

All statistical analyses were performed using the SPSS16.0 statistical software package (Version 16.0, SPSS Inc., Chicago, IL). The χ^2^ test was used to examine the differences in demographic variables and the distributions of genotypes between cases and controls. Odds ratios (OR) and 95% confidence intervals (CI) were used to evaluate the association of *XPF* variants with the risk of ESCC by unconditional logistic regression model adjusted by age, sex and smoking status. All statistical tests were two-sided tests, and a *P* value of <0.05 was considered significant.

## Results

### Subject characteristics

The genotype distributions of select characteristics of participants in this study were shown in Table 1. There were no statistically differences between cases and controls for Tangshan case-control set and Beijing case-control set in terms of age and sex distribution (all *P*>0.05), indicating that frequency matching was adequate. However, there were more smokers among patients compared with controls in both case-control sets (Tangshan set: 61.2% *vs* 29.2%, *P*<0.001; Beijing set: 65.5% *vs*32.3%, *P*<0.001).

**Table 1:** Distribution of select characteristic among ESCC cases and controls.

Variables	Tangshan case-control set					Beijing case-control set				
	Cases		Controls		*P* value*	Cases		Controls		*P* value*
	No (%)		No (%)			No (%)		No (%)		
Total	500		500			1024		1024		
Sex					1.000					1.000
Male	404 (80.8)		404 (80.8)			837 (81.7)		837 (81.7)		
Female	96 (19.2)		96 (19.2)			187 (18.3)		187 (18.3)		
Age					1.000					1.000
<50	74 (14.8)		74 (14.8)			134 (13.1)		134 (13.1)		
50–59	170 (34.0)		170 (34.0)			338 (33.0)		338 (33.0)		
60–69	172 (34.4)		172 (34.4)			406 (39.6)		406 (39.6)		
≥70	84 (16.8)		84 (16.8)			146 (14.3)		146 (14.3)		
Smoking status					<0.001					<0.001
Non-smoker	194 (38.8)		354 (70.8)			353 (34.5)		693 (67.7)		
Smoker	306 (61.2)		146 (29.2)			671 (65.5)		331 (32.3)		

*Two-side χ2 test.

### *XPF* variants and risk of ESCC

The genotype distributions of *XPF*-673C>T and 11985A>G polymorphisms in the cases and controls were summarized in Table 2. The observed genotype frequencies of *XPF* polymorphism (-673C>T and 11985A>G) in both controls were consistent with Hardy-Weinberg equilibrium in both sets (Tangshan set: *P* = 0.06 and *P* = 0.50; Beijing set: *P* = 0.40 and *P* = 0.97).

**Table 2:** Genotype frequencies of *XPF* variants among patients and controls and their association with ERCC risk.

Genotypes	Cases		Controls		*OR* (95% *CI*)*	*P* value
	No	%	No	%		
-673C>T						
Tangshan	N = 500		N = 500			
CC	297	59.4	277	55.4	1.00 (Ref)	
CT	175	35.0	176	35.2	0.97 (0.73–1.29)	0.850
TT	28	5.6	47	9.4	0.58 (0.34–0.99)	0.040
Beijing	N = 1024		N = 1024			
CC	597	58.3	547	53.4	1.00 (Ref)	
CT	363	35.4	391	38.2	0.83 (0.68–1.01)	0.068
TT	64	6.3	86	8.4	0.66 (0.46–0.95)	0.027
Total	N = 1524		N = 1524			
CC	894	58.7	824	54.1	1.00 (Ref)	
CT	538	35.3	567	37.2	0.88 (0.75–1.04)	0.129
TT	92	6.0	133	8.7	0.64 (0.47–0.86)	0.004
11985A>G						
Tangshan	N = 500		N = 500			
AA	315	63.0	318	63.6	1.00 (Ref)	
AG	169	33.8	156	31.2	1.13 (0.85–1.50)	0.420
GG	16	3.2	26	5.2	0.61 (0.31–1.21)	0.160
Beijing	N = 1024		N = 1024			
AA	642		658		1.00 (Ref)	
AG	339		324		1.10 (0.90–1.35)	0.342
GG	43		42		0.97 (0.61–1.55)	0.900
Total	N = 1524		N = 1524			
AA	957	62.8	976	64.0	1.00 (Ref)	
AG	508	33.3	480	31.5	1.11(0.95–1.31)	0.199
GG	59	3.9	68	4.5	0.84 (0.57–1.23)	0.361

*Data were calculated by unconditional logistic regression, with the CC genotype as reference group and adjusted for gender, age, and smoking status.

Multivariate logistic regression analysis were used to calculate the association of XPF -673C>T or 11985A>G genotypes with ESCC risk (Table 2). For -673 C>T polymorphism, TT genotype was shown to be a protective genotype. Compared with -673CC genotype, -673TT genotype was related to a decrease risk of ESCC in Tangshan case-control set (OR = 0.58, 95% CI = 0.34–0.99, *P* = 0.04). Similarity, logistic regression analyses revealed that individuals with -673TT genotype were also had a decreased risk for ESCC with OR (95%CI) of 0.66(0.46–0.95) in Beijing case-control set. In the pooled analyses, we found that -673TT genotype carriers had a 0.64-fold decreased risk to develop ESCC (95%CI = 0.47–0.86). For 11985 A>G polymorphism, our study didn't show any association of genotypes of 11985A>G polymorphism with the risk of ESCC.

The risk of ESCC associated with the -673C>T polymorphism was further evaluated by stratifying for age, sex and smoking status using the combined data of two case-control sets (Table 3). In stratified analyses with age, -673TT genotype was significantly associated with decreased risk among subjects aged 60 years or younger (OR  = 0.54, 95% CI  = 0.36–0.80, *P*  = 0.002), but not among subjects aged older than 60 years (OR  = 0.81, 95% CI  = 0.51–1.28, *P*  = 0.359). There was a significant gene-age interaction was observed *(P* for interaction = 0.002). Compared with the -673CC genotype, a significantly decreased risk of ESCC was associated with TT genotypes only among males (OR  = 0.67, 95%CI  = 0.48–0.93, *P*  = 0.017), but not among females (OR  = 0.58, 95%CI  = 0.28–1.21, *P*  = 0.145). There was a significant gene-sex interaction (*P* for interaction = 0.002).

**Table 3:** Association of *XPF* -673C>T variant with ESCC risk stratified by selected variables.

Variable	Genotypes (Cases/Controls)					TT/CC model *OR* (95% *CI*)*	*P* value	*P*_interaction_ §
	CC		CT		TT			
Sex								
Male	724/678		439/453		78/110	0.67 (0.48–0.93)	0.017	0.002
Female	170/146		99/114		14/23	0.58 (0.28–1.21)	0.145	
Age								
≤60	455/439		299/259		48/83	0.54 (0.36–0.80)	0.002	0.002
>60	439/358		239/308		44/50	0.81 (0.51–1.28)	0.359	
Smoking status								0.002
Non-smoker	321/561		196/396		30/90	0.61 (0.39–0.96)	0.031	
Smoker	573/263		342/171		62/43	0.66 (0.43–1.00)	0.050	

* Data were calculated by unc°nditional logistic regression and adjusted for gender, age, and smoking status, where it was appropriate.

§ *P* values for gene-environment interaction.

Because tobacco smoking is predisposing factor for ESCC, we then investigated whether a gene–smoking interaction existed between the -673C>T polymorphism and smoking (Table 3). Among nonsmokers, compared with the -673CC carriers, individuals with TT genotype had a 0.61-fold decreased risk to develop ESCC (95% CI  = 0.39–0.96, P = 0.031). Among smokers, there was marginally significantly decreased ESCC risk (OR = 0.66, 95% CI  = 0.43–1.00, P = 0.050) for individuals with TT genotype compared with those with AA genotype. A multiplicative gene–smoking interaction was also found with *P* for interaction equaling to 0.002.

## Discussion

In this study, we investigated the associations of *XPF* -673C>T and 11985A>G genetic variants with the risk of ESCC in Chinese population. We found that subjects with -673TT genotype decreased the risk of ESCC compared with -673CC genotype carriers in Chinese population. For 11985A>G polymorphism, there was no significant difference between ESCC cases and controls.

The ethnic difference in the *XPF* polymorphisms might have significant effect on disease phenotype. Researchers found a significant association of *XPF* rs1799801 in exon 11 with a reduced risk of bladder cancer in Caucasian population [18], but not in Chinese population [15]. This discrepancy may reflect the difference of genetic background among different study populations. In present study, the genotype frequencies of the *XPF*-673C>T in controls are 59.4% for CC, 35.0% for CT and 5.6% for TT, which are consistent to those in Yu's study (55.5%for CC, 36.7% for CT, 7.8% for TT) and those in Shao's study (57.3% for CC, 37.4% for CT, 5.2% for TT) [13], [16]. To our knowledge, no reported data on the 11985A>G polymorphisms are available.

XPF is an essential protein in NER pathway, which is responsible for removing DNA adducts induced by platinum based compounds [19]. As a structure specific DNA endonuclease, XPF has been reported to bind to double-stranded DNA [20]and further participated in several DNA repair pathways by combining with ERCC1 [21], [22]. As a crucial rate-limiting factor in NER, the low expression of XPF acted as a genetic susceptibility factor in the development of cancers and a prognosis risk factor after chemotherapy in several of cancers, including esophageal cancer [15], [23], [24], [25]. The functional analysis of *XPF* -673T>C showed that -673T allele had a significantly higher transcriptional activity compared with the -673C allele. The epidemiology experiments also showed that -673TT genotype had a decreased risk of lung cancer compared with the -673CC genotype [13], [16]. These results further supported our present results, which showed the carriers with -673TT genotype had a significantly decreased risk of esophageal cancer.

Multiple evidences have revealed statistically significant gene-environment interaction in various cancers [26], [27], [28]. Most ESCC occurred also due to interactions between environmental and genetic factors [2], [4]. NER pathway is the primary mechanism for the pair of bulky and helical distorting DNA adducts generated by cigarette [29], [30]. Tobacco smoke contains many of carcinogens and procarcinogens, such as benzo(a)pyrene and nitrosamine. Genetic variant in the promoter of *XPF* may influence the activation of substrates in cigarette smoke and then contribute to the different susceptibility to cancers. Several studies have evaluated the relationship between *XPF* -673C>T polymorphism and lung cancer susceptibility by smoking status. A study conducted by Shao showed that -673 TT or CT genotype significantly increased the risk of lung cancer in non-smokers, but not in smokers [16]. However, Yu and his colleagues didn't found any interaction between -673C>T polymorphism and smoking status [13].This is the first report for showing the gene-smoking interaction between *XPF* genotypes and smoking status in ESCC risk. Our results suggested that XPF -673TT genotype was related to a decreased ESCC risk among non-smokers, but not among smokers. As a key DNA damage repair protein, the low expression of XPF can delay DNA repair, and further increase genome instability and promote tumorigenesis. However, a large mount of exposure to tobacco smoking may overwhelm the difference of the DNA repair capacity by different genotype.

Our study has its limitation. This study is a hospital-based case-control study restricted on Chinese Han population. Selection bias may arise when the sampling is not random within the subpopulations of cancer and cancer-free subjects. The controls in our study were matched to the cases by age and sex, which might minimize the subject selection bias. In addition, there were several of *XPF* polymorphisms were discovered and have been demonstrated to be associated with various cancers. More potential functional XPF polymorphisms still need to be validated by larger studies with diverse populations.

In conclusion, *XPF* -673C>T polymorphism was significantly associated with a decreased risk of ESCC. Our finding suggests that *XPF* play a role in the etiology of ESCC.
